# Polarisation optics for biomedical and clinical applications: a review

**DOI:** 10.1038/s41377-021-00639-x

**Published:** 2021-09-22

**Authors:** Chao He, Honghui He, Jintao Chang, Binguo Chen, Hui Ma, Martin J. Booth

**Affiliations:** 1 Department of Engineering Science, University of Oxford, Parks Road, Oxford, OX1 3PJ UK; 2 Guangdong Engineering Center of Polarisation Imaging and Sensing Technology, Tsinghua Shenzhen International Graduate School, Tsinghua University, 518055 Shenzhen, China; 3 Institute of Biopharmaceutical and Health Engineering, Tsinghua Shenzhen International Graduate School, Tsinghua University, 518055 Shenzhen, China; 4 Department of Physics, Tsinghua University, 100084 Beijing, China; 5 Department of Biomedical Engineering, Tsinghua University, 100084 Beijing, China

**Keywords:** Biophotonics, Applied optics

## Abstract

Many polarisation techniques have been harnessed for decades in biological and clinical research, each based upon measurement of the vectorial properties of light or the vectorial transformations imposed on light by objects. Various advanced vector measurement/sensing techniques, physical interpretation methods, and approaches to analyse biomedically relevant information have been developed and harnessed. In this review, we focus mainly on summarising methodologies and applications related to tissue polarimetry, with an emphasis on the adoption of the Stokes–Mueller formalism. Several recent breakthroughs, development trends, and potential multimodal uses in conjunction with other techniques are also presented. The primary goal of the review is to give the reader a general overview in the use of vectorial information that can be obtained by polarisation optics for applications in biomedical and clinical research.

## Abstract (web summary)

The review focuses on methodologies and biomedical applications of polarisation optics. It also presents prospects on development trends, the potential multi-modal uses in conjunction with other techniques.

## Graphical abstract

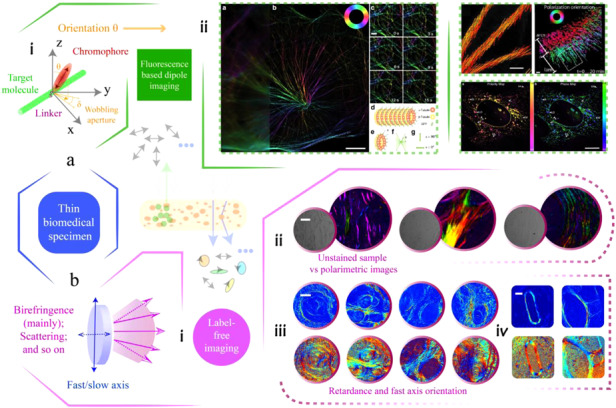

## Introduction

**Fig. 1:**
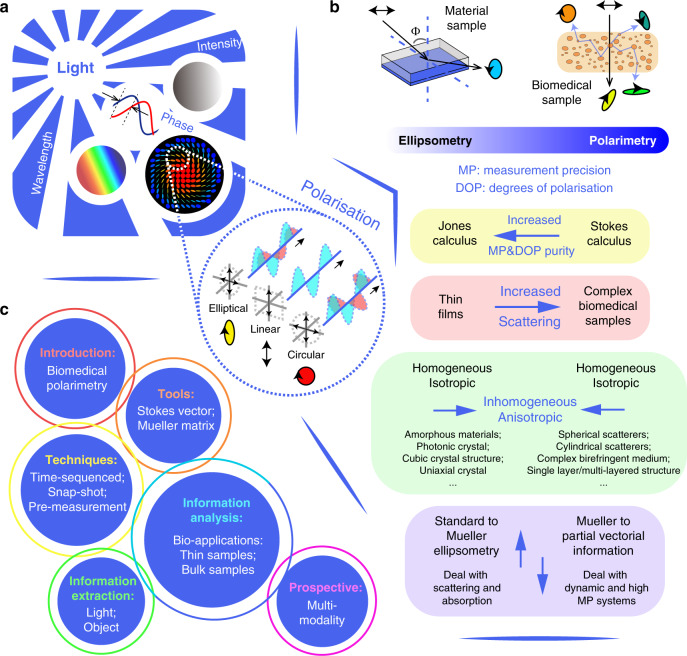
Developing trends of tissue polarimetry and the structure of this review. **a** Light properties: intensity, wavelength, phase and polarisation. **b** The comparisons of the development of modern ellipsometry and polarimetry. The inset blue arrows in different colour boxes represent either changing directions or developing trends. **c** The structure of this review

Light, as an electromagnetic wave, possesses several fundamental properties, which include intensity, wavelength, phase and polarisation^1,2^ (see Fig. 1a). While the former three are scalar quantities, polarisation has vectorial properties; its use has therefore required more advanced optical and computational approaches. Hence, studies of either the vector properties of light, described via the state of polarisation (SOP) or the full vectorial transformation properties of an object, have a shorter history in biomedical analysis compared with their scalar counterparts, and the extent of their application is still being explored^3–5^. So far, numerous intriguing areas of research have been enhanced through harnessing vectorial information acquired via polarisation optics; these range from fundamental research^6–10^, such as quantified polarisation entropy^11^, across quantum physics^12^, such as spin-orbital interaction of light^13,14^, to material characterisation (e.g. chiral characteristics^15^) or for biomedical studies and clinical applications (e.g. characterisation of structural features in tissue^16–21^).

Scattering, especially through multiple-scattering processes, alters the degree of polarisation and SOP of the incident light beam^22^. While it is an insightful procedure for evaluating structural information of biomedical samples including tissues and cells^16^, it also introduces uncertainty in expected photon properties^22^. This characteristic largely hinders the development of modern tissue polarimetric techniques and related information analysis^20,22,23^. The turbidity of many tissue structures imposes randomness on the photons’ interaction processes, which complicates the detection and analysis of vectorial information^20^. Such phenomena also distinguish tissue polarimetry from the traditional polarisation measurement technique of ellipsometry^22–26^. As summarised in Fig. 1b, their comparison shows several commonalities and differences. The Jones formalism is used for clear and non-depolarising media such as thin films; it consists of the Jones vector (describing the polarisation property of the light) and Jones matrix (describing the polarisation transformation properties of the object). They have been widely used in ellipsometry techniques^25,26^ (see Fig. 1b; and summary in ref. ^26^). Another polarisation formalism is Stokes–Mueller, in which the Stokes vector and the Mueller matrix are used to describe the light beam and the object, respectively. Neither the Stokes vector nor the Mueller matrix maintain absolute phase information, but have the advantage of being able to represent depolarisation^27,28^. This is often essential in biomedical polarimetry, whose applications normally involve scattering induced light depolarisation^20–23^. There exists an increasing trend in both modern ellipsometry and polarimetry to deal with increasingly complex media, moving from isotropic and homogeneous media towards anisotropic and inhomogeneous ones^20–31^. While modern ellipsometry is developing towards full polarisation measurement using the Stokes–Mueller formalism, advanced polarimetry is gradually changing from full vectorial measurement to partial detection, as some key features of biomedical specimens could possibly be revealed through partial, rather than complete, measurements of vectorial information^16,32,33^.

The structure of this review is given in Fig. 1c; it consists of introducing the basic polarisation optical tools, summarising the current vectorial information detection, extraction, and analysis approaches, and pointing out the possibilities for future multi-modal synergy with other cutting-edge technologies. Although biomedical polarimetry is still developing towards various research fields and applications, largely unexplored spaces still exist. We also hope this review could stimulate new explorations or breakthroughs in such prospective fields.

It is worth noting that the use of biomedical polarimetry is expanding and has also been summarised in several recent reviews by Tuchin^22^, Ghosh & Vitkin^20^, Ramella-Roman et al.^23^, Qi & Elson^24^, De Boer et al.^34^, He et al.^16^. They have demonstrated the fast progress of this technique in the biomedical and clinical fields.

## Fundamental vectorial representation for polarised optics in biomedical applications

**Fig. 2:**
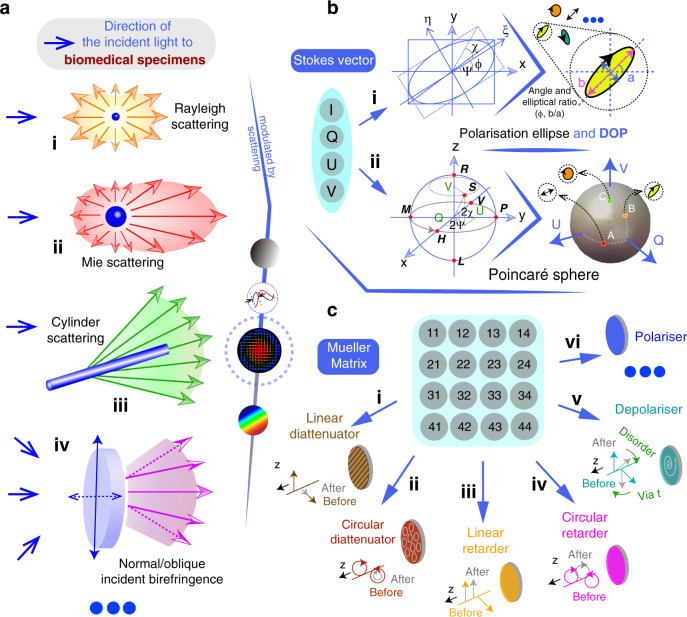
Fundamental light-matter interaction processes in biomedical specimens and polarisation tools. **a** Several scattering types and media manipulation, showing schematically the way in which light is scattered in the differing scenarios^16–24^. **b** The Stokes vector representations. Top row: the polarisation ellipse and the DOP can be used to represent a given Stokes vector. Bottom row: the Poincaré sphere visualises all states of polarisation with linear states on the equator, circular states on the north/south poles and elliptical states in between^2–5^. **c** Vectorial properties that can be encoded in the Mueller matrix. Linear/circular diattenuator, linear/circular retarder etc. are fundamental polarisation elements; the arrows on the lines or circles represent the eigenbasis of modulated light beam passing through such media, where ‘before’ and ‘after’ illustrate the amplitude and/or relative phase change of the chosen eigenbasis. For further details refer to refs. ^2–5^

Sample induced scattering is prevalent in biomedical imaging, particularly in tissues^16,20,22,23^. This introduces additional SOP modulations that affect diattenuation and retardance as well as depolarisation^16,22,23^. A complex scattering medium can often be modelled by several basic components, like spherical scatterers of different sizes^35^; cylindrical rod-like scatterers with different orientational distributions^36^; and birefringence for an interstitial medium^37–39^; combinations of these features can all be adjusted to mimic the real object^36,40–42^. Other physical conditions such as layer complexity (single-layered or multi-layered scattering^43^), or scattering type (elastic domain for Mie and Rayleigh scattering; or inelastic domain, like Raman scattering) are also described in the literature, e.g., see ref. ^44^. Modelling of the scattering assumptions can be conducted via Monte Carlo simulation^45,46^. This is a widely used statistical method for quantitative analysis of the interactions between polarised photons and complex biomedical media^40–42^, especially bulk media with multiple-scattering properties, for which the analytical solutions to describe the interactions cannot be obtained. In this review, we focus on the occurrence of elastic scattering in conjunction with other polarisation characteristics (see Fig. 2a) for biomedical polarimetry.

In the presence of depolarisation, Jones calculus, which represents only transitions between pure polarisation states, is of limited use as it cannot comprehensively describe the light properties, especially the degree of polarisation for partially polarised light^2–5^. Intrinsically, Jones calculus is based on the assumption that electric field vector holds a particular stationary state. For partially polarised light (or fully depolarised light), the variation of the electrical vector as the light propagates is semi-disordered (or completely disordered) so that more degrees of freedom are required to describe the light field^47^. In the scope of linear optics, the Stokes vector, which is a 4 × 1 vector, is used to characterise the SOP of the light beam^47,48^; while the Mueller matrix, which is a 4 × 4 matrix, describes the transformation properties of the object that affect the Stokes vector^47,48^. Hence, considering that the scope of this review focuses on tissue polarimetry, we place an emphasis on the Stokes–Mueller formalism.

### Stokes vector

The Stokes vector can be expressed with the format shown in Fig. 2b^47,48^; where

$$I = I_0 + I_{90}$$

$$Q = I_0 - I_{90}$$

$$U = I_{45} - I_{ - 45}$$

$$V = I_R - I_L$$

$I_0$, $I_{90}$, $I_{45}$, $I_{ - 45}$ are the projection intensities (different linear components in directions of 0°, 90°, 45°, −45° with respect to the local coordinate system) of a light beam, $I_L$ and $I_R$ are components of left/right-handed circular polarised light, respectively. Note some other parameters can be defined with components of Stokes vector: degree of polarisation

$${\rm{DOP}} = \sqrt {Q^2 + U^2 + V^2} /I$$

degree of linear polarisation

$${\rm{DOLP}} = \sqrt {Q^2 + U^2} /I$$

and degree of circular polarisation of light^3–5^.

$${\rm{DOCP}} = \sqrt {V^2} /I$$

From the above expressions, we see that the Stokes vector can be calculated via intensity measurements that can be readily performed in an experiment^47,48^. The Jones vector, on the other hand, is defined by amplitude and phase that cannot be directly measured, which is another reason why the Jones approach is less well suited to biomedical polarimetry^20–23^. The intrinsic reason for the existence of depolarisation is due to temporal or spatial averaging^16,20–23^. If an extremely fast and small detector could monitor the vector properties of the light, then it would only detect polarised light. Such averaging properties can also be found in the definition of the Stokes vector^47,48^. Note the definitions of right-handed circular polarised light (clockwise rotation) and left-handed circular polarised light (anticlockwise rotation) are different in optics books and academic communities. It depends on whether the observer ‘sees’ the light from the source (Convention I), or from the detector (Convention II). Institute of Electrical and Electronics Engineers (IEEE) uses Convention I, so it is also widely used in engineering fields; Quantum physicists also use Convention I, to be consistent with the conventions for representing particle spin states^49,50^. However, for numerous optics books such as Principles of Optics (Born and Wolf^48^) and Handbook of Optics^51^, Convention II is used. In this review, we use Convention II in order to correspond to such scientific references.

The Jones vector has a graphical representation known as the polarisation ellipse^47,48^ (if we add the parameter DOP, the polarisation ellipse can also represent the Stokes vector (see Fig. 2b (i))). While for Stokes vector visualisation, the Poincaré sphere (PS) is commonly used^47,48^ (see Fig. 2b (ii)). SOPs are represented via the PS, which is defined in a three-dimensional coordinate system, whose coordinates correspond to the eigenbasis formed by $Q$, $U$ and $V$ (each normalised by $I$). The PS is a unitary sphere that represents complete polarisation states on its surface and depolarised states inside the sphere. Any transformation of the SOP through a specimen is equivalent to manipulation of the original Stokes vector between different points on or inside the PS. Figure 2b (ii) gives a schematic demonstration of the PS. The length of the vector from the origin point to the SOP location denotes the DOP^47,48^. The letters $H$, $V$, $M$, $P$ are specific polarisation states: horizontally polarised ($H$), vertically polarised ($V$), 45° polarised ($M$) and −45° polarised ($P$). The polarisation ellipse parameters (*χ* and *ψ*) can be interpreted from the azimuth angle (and the polar angle) of the derived vector inside the PS.

Such a graphical representation excludes the absolute phase information, which is sometimes not addressed in typical vectorial beam analysis such as pure polarisation measurement or tissue polarimetry^20–23,47,48^. However, one type of the absolute phase variation that is referred to as geometric phase is related to the pathway of the SOP movement on the surface of the PS^47,48^, which features scope for the extension of the tissue polarimetric technique (also see “Discussion”).

### Mueller matrix

The Mueller matrix (MM) describes the vectorial transformation properties of an object^16,20–23^. As illustrated in Fig. 2c, the MM describes the transformation of one Stokes vector into another. The MM represents the full vector properties of an object through its 16 elements ($m_{kl};k,l = 1,2,3,4$). Among these, $m_{11}$ represents the transformation of scalar intensity (absorption or other loss); the other 15 elements encode the vectorial properties of the object^47,48^. Direct physical meanings of these 15 elements taken individually are normally ambiguous^16,20–23^. As illustrated in Fig. 2c, several fundamental polarisation properties are encoded in (and can be extracted from) the MM. They are linear/circular diattenuation, linear/circular retardance, linear/circular polarisance, linear/circular depolarisation and so on^2–5,52–62^. The effects of each of these fundamental optical mechanisms on the light vectors along the propagation direction z are shown in Fig. 2c (i) to (vi), where ‘before’ and ‘after’ illustrate the amplitude and/or relative phase change of the chosen eigenbasis. Amongst these mechanisms, the diattenuator possesses two different absorption ratios for two polarisation directions; it in effect reduces the intensity of one polarisation compared to the other. The retarder exhibits different refractive indices for two polarised eigenvectors, in effect leading to an additional relative phase difference between the two vectors. The depolariser can modify the DOP of the light beams. For more detailed descriptions of the mechanisms and further examples see refs. ^2–5^.

Both Stokes vectors and MMs can represent the effects of time-averaged induced depolarisation^16^. An object may introduce two different classes of depolarisation: homogenous depolarisation and inhomogeneous depolarisation. The former one can lead to a similar DOP change for any SOP; such properties can be observed in media such as a polystyrene sphere solution. The later one can lead to different DOP change for different SOP; typical examples are found in complex biomedical tissue.

Several factors may contribute to depolarisation in experimental scenarios. We describe three main reasons here. (a) The first reason relates to the time domain. In general, the Stokes vector polarimeter is based on intensity measurement^26^, so in practice the intensity recorded at the detector includes a time-integration process. If the SOP changes rapidly, possibly due to multi-scattering induced by complex bio-media, then depolarisation would be measured. (b) This reason relates to the spatial domain. When imaging processes are involved, every point on the beam section is created through the integration of various sub-beams that could have different polarisation states. The superposition of these states leads to depolarisation. (c) The final reason is given in the spectral domain. Many processes that affect polarisation, such as birefringence and scattering, are also dependent on wavelength. Hence for different wavelengths, variations in amplitude and phase may also lead to depolarisation.

## Vectorial information measurement techniques for biomedical applications

**Fig. 3:**
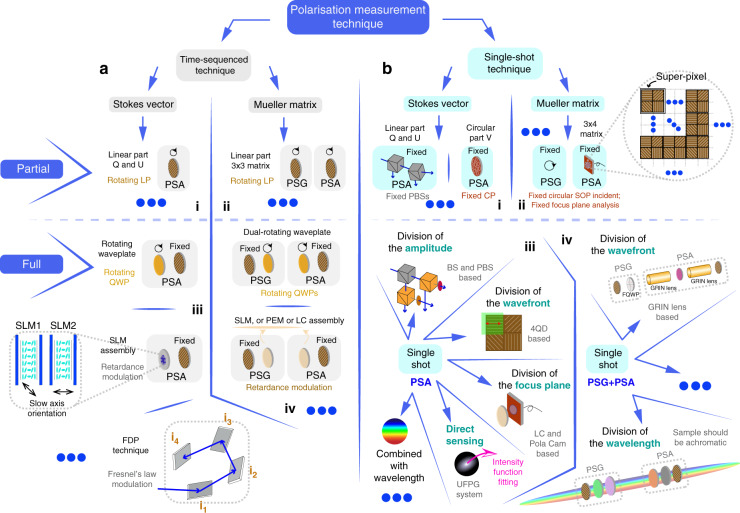
Time-sequenced and single-shot polarisation measurement techniques. **a** Time-sequenced techniques: (i) Partial Stokes vector polarimetry; the PSA can be a rotating polariser. (ii) Partial MM polarimetry; the PSG and PSA can both be a rotating polariser. (iii) Full Stokes vector polarimetry; the PSA and PSG can both be a tuneable retarder (rotating quarter waveplate or SLM assembly) followed by a fixed polariser^28,63,79^. (iv) Full MM polarimetry; the PSG can be a fixed polariser followed by a tuneable retarder^28,63,79^ (rotating quarter waveplate or LC components); the PSA can be a tuneable retarder (rotating quarter waveplate or LC components) followed by a fixed polariser^28,63,79^. **b** Snap-shot techniques: (i) Partial Stokes vector polarimetry; the PSA can be a fixed PBS assembly or circular polariser. (ii) Partial MM polarimetry; the PSA can be a fixed polariser array^32^. (iii) Full Stokes vector polarimetry; the types consist of division of the amplitude^97–99^, division of the wavefront^71,100–105^, division of the focus plane^106–122^, and so on. (iv) Full MM polarimetry; the types consist of division of wavefront^72^, division of the wavelength^67,96^, and so on. (LP: linear polariser; QWP: quarter waveplate; FDP: four-detector photopolarimeter; for more information about such Fresnel’s law based polarimetry refer to ref. ^288^; BS: beam splitter; PBS: polarisation beam splitter; CP: circular polariser; FQD: four quadrant detector; FQWP: four quadrant waveplate; UFPG: universal full Poincaré generator)

**Fig. 4:**
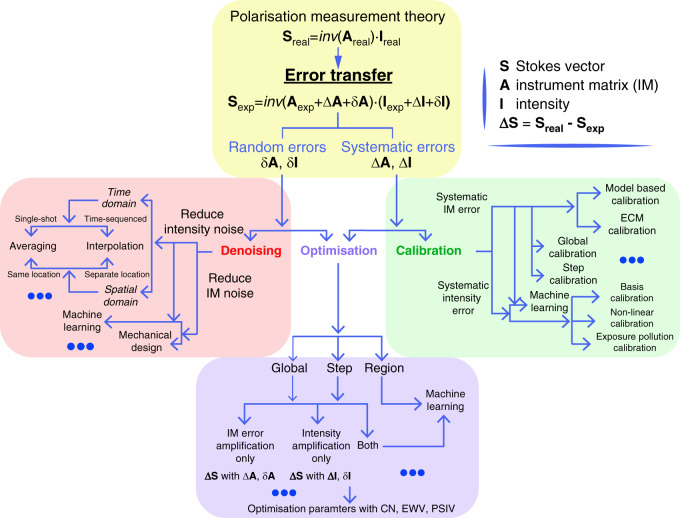
Polarisation measurement theory for Stokes vectors. The polarisation measurement theory is summarised in three aspects: denoising, optimisation, and calibration

Numerous vectorial information measurement methods have been put forward in the past decades^4,7,11,26,28,29,63^. In this section, we categorise the polarisation measurement techniques into two types: time-sequenced and snap-shot approaches^28,29,64–67^ (see Fig. 3). For both cases, the preparation required before detection is similar and can be divided into three general steps: denoising, optimisation and calibration^32,68–78^ (see Fig. 4). The aim of those steps is to reduce the complex errors that would occur during the measurement process, hence obtaining imaging results with higher precision and accuracy^32,69–72^. The technical aspects of such advanced polarimetry are summarised in the review papers by Azzam^28^, Chipman^63^ and Tyo^79^.

Before full Stokes vector/MM measurements became widely adopted, there was successful work using fixed input SOP and fixed analysers to perform partial vectorial detection for biomedical applications. Jacques et al. showed crossed-polarised light imaging to enhance surface contrast, detect skin cancer and other lesion margins^80,81^; Demos et al. added the dimension of wavelength based on crossed SOPs^82,83^; Groner et al. noted such techniques can enhance superficial vascular contrast, and hence adopt it into brain perfusion, pancreatic and further clinical diagnoses^84^; Bargo et al. took angle-dependency into consideration when measuring skin tissue^85^. Sridhar et al. also studied multiply scattered photons to enhance information extraction from biological specimens via elliptically polarised light^86^.

Both time-sequenced and snap-shot polarimetry techniques can be classified in two general ways: firstly, as either Stokes vector (light property) or MM (material property) measurement; and secondly, as partial or full vectorial measurement (Fig. 3). We will classify different techniques using the second criteria in later sections of this review.

### Time-sequenced techniques

Stokes polarimetry is clearly the basis for more advanced MM polarimetry. Both of their intrinsic mechanisms can be interpreted with respect to the instrument matrix (*A*)^68–72^ (see Fig. 4). This matrix represents the settings of the polarisation state generator (PSG) and polarisation state analyser (PSA) in the various measurement steps: for MM measurement it represents the PSG and PSA, for Stokes vector measurement it represents one the PSA. Combinations of the rotating waveplate and/or polariser are widely adopted in such approaches^64–66^. The original proposal for a Stokes vector measurement scheme (that using SOPs of $H$, $V$, $M$, $P$, $L$ and $R$) was from Collett^87^ in 1984. Later it was adopted for biomedical information extraction or phantom analysis with ability of the full depolarisation information characterisation^16,20–23^.

The use of rotating components has disadvantages, such as increasing measurement time and introducing unexpected errors from mechanical movements. However, such systems are easy to construct. Hence, numerous commercialised polarimeters still use this approach. In order to make improvements, researchers have tried to reduce the number of the rotating components (such as the dual-rotating waveplate MM polarimeter with fixed polarisers that was proposed by Azzam^64^ in 1978, which is widely used in tissue analysis^16,22^) or use fast modulation devices (such as Stokes or MM polarimeters enabled via liquid crystal variable retarders (LCVR)^88^, spatial light modulators (SLM)^89^, ferroelectric liquid crystals (FLC)^90^, or photoelastic modulators (PEM)^91^). Besides full MM detection, partial MM measurement, such as 3 × 3 MM imaging of linear polarisation states, also gained wide attention. Qi et al. used related methods in analysing linear depolarisation and retardance of rat tissue^92^.

Although there are some applications that require high-speed operation, such as detection in dynamic situations like in vivo sensing for clinical diagnosis^24^, time-sequenced polarimeters still play an important role in modern polarimetric research, due to their mature state of development and simple configuration. Such applications include characterisation of complex vector fields^6,7,9^, or providing ground truth validation in tissue research (e.g., differentiating human breast cancer^93–95^).

### Snap-shot techniques

Rapidly changing or dynamic objects need snap-shot detection, in order to correctly extract vectorial information that would be complicated by time-sequenced measurement. Snap-shot approaches are configured to take different measurements in parallel, as opposed to the serial measurement of sequential techniques. In general, snap-shot techniques must, to some degree, sacrifice alternative dimensions to enable simultaneous vector measurement^67,96^. Those methods include (see Fig. 3b): Stokes vector polarimeters with division-of-amplitude^97–99^, division-of-wavefront^71,100–105^ or division-of-focus-plane^106–122^— these fit in the category of spatial modulation with respect to different analysis channels (see Fig. 3b (iii)). Savart-plate-based polarimeters (Oka et al.) are in the category of Fourier frequency domain segmentation, which are interferometric systems where the polarisation information is encoded in the spatial carrier fringes^123^. If combined with the property of birefringence dispersion, spectroscopic polarimetry with channelled spectrum can also be presented^67,96^.

Similar to Stokes vector polarimeters, there exist concepts for snap-shot MM polarimeters, in which certain dimensions are sacrificed to enable simultaneous MM estimation (see Fig. 3b (iv)). Dubreuil et al.^67^ and Hagen et al. ^96^ utilised different wavelength-dependent birefringent media to resolve the MM in a single shot, within the limitation of the sample being achromatic. Piquero et al. ^124^ utilised full Poincaré beams as a PSG, enabling MM polarimetry with the division of the wavefront. He et al. applied a spatially segmented method with defocusing to measure statistically averaged properties of biomedical samples^72^. As complete snap-shot MM techniques are rather complex, their usage for extractions of biomedical information is less common than the use of single-shot partial MM or Stokes vector polarimetry. For instance, 3 × 4 MM imaging is also gaining attention using circularly polarised illumination^32^; Chang et al. brought such a technique into the human liver and cervical carcinoma tissue analysis^32^ (see Fig. 3b (ii)).

### Denoising, optimisation and calibration

The measurement precision and sensitivity are vital in polarimetric techniques, hence the errors need to be properly controlled^28,32,68–78,125^. However, as Stokes vector or MM measurements belong to high dimensional information detection with multiple components^64,66,126^, the error sources and error transfer process (such as accumulated amplifications through matrix calculations) are very complicated. Several previous analyses can be found in refs. ^28,29,32,68–78,125–128^. In Fig. 4, we summarise in one diagram an overview of the structure of the ‘denoising, optimisation, and calibration’ processes in polarimetric techniques with respect to random errors (δ**A**, δ**I**) and systematic errors (Δ**A**, Δ**I**). It also can be seen in the figure that three directions towards obtaining the correct vectorial measurements are still developing.

Note again that **A** is the instrument matrix for polarimetric measurement specifically, which is determined by the systematic configurations of polarisation optics and determines the error propagation amplification^20–24,69–72^; while **I** refers to the recorded intensity information. In Fig. 4, we take Stokes vector measurement equation (**S** = inv(**A**)·**I**)^20–24,69–72^ as a main illustration, to show the relationships between three steps in a picture, for simplicity. A similar structure (using the generalised equation: **M** = inv(**A′**)·**I**) can be derived for the MM measurement, which is based fundamentally on the Stokes vector measurement process.

In order to reduce δ**A** and δ**I**^32,68–78^, a ‘denoising process’ is adopted. Figure 4 shows the approaches in time or spatial domain including time average and interpretation methods. To deal with the Δ**A** and Δ**I**, a ‘calibration process’ is required. Numerous polarimetric calibration methods have been proposed^23,29,63,71,129^; these can be divided into global and local calibration approaches. Note that the calibration process itself also suffers from the error transfer process. Hence, determining the SOPs for calibration, choosing the standard calibration samples, as well as designing specific calibration methods for different systems should be taken into consideration^130,131^. Figure 4 also shows the process of ‘optimisation’, which can deal with both types of errors, through global and local optimisation approaches. For this process, different evaluation standards have been put forward to estimate the systematic performance. Marenko et al. considered the condition number (CN) in polarimetric optimisation^132^, Ambirajan, Tyo and others have analysed CN-based optimisation on different phantoms^70,133–137^; and Sabatke et al. introduced equally weighted variance (EWV)^69^ into the polarimetric area; Azzam et al. and following researchers explained the usage of geometry optimisation based on Poincaré sphere internal volume (PSIV)^138–140^. Other useful criteria have also been proposed^141,142^. Such optimisation parameters can be used for evaluating the intrinsic error amplification of a polarimetry, which affect the accuracy and precision of the measurement^23,29,63,71,129–131^. If we consider the CN, the minimum CN value for a matrix-based Stokes polarimetry is $\sqrt 3$, which is the theoretical limit for systematic error amplification^68,70^, as opposed to the minimum possible CN value (CN = 1) for matrix inversion. A similar error amplification also exists in MM polarimetry^143^. The three above-mentioned processes (denoising, optimisation, calibration) are vital for any biomedical polarimetry, as they determine the credibility of the information extraction and further analysis.

For the matrix-based calculation of Stokes polarimetry (within the scope of the above explanations), there exist two problems: first, the mathematical aspect of minimal error amplification through the matrix calculation; second, the practical aspect that the above-mentioned three separate procedures contribute to error accumulation separately, as they require different evaluation criteria and are normally based upon different assumptions. In fact, there exists the possibility to jump out of the domain of matrix calculation for Stokes polarimetry, circumventing those drawbacks. An interesting direction is the adoption of a full Poincaré beam, taking advantage of its feature that maps all SOPs in a single beam^144^. Vella, Zimmerman, He and others have proposed different measurement approaches harnessing such beams based on different phantoms such as stress engineered optics^145–151^ and graded index optics^68^. The full Poincaré beam Stokes vector technique has recently made it possible to have a clear information-based learning approach (such as the task of searching for the brightest points), combining the ‘end to end’ solution (a combination of above three processes—denoising, optimisation and calibration) together for an enhanced polarimetric measurement precision and accuracy^68^. In essence, this approach means that the Stokes vector retrieval process changes from matrix-based calculation to information-based image processing.

## Vectorial information extraction methods for biomedical applications

Information about the vectorial properties of a biological specimen can be derived partially from the polarisation properties of the light beam or, in a more complete fashion, from the polarisation properties of the tissue itself^20–23,152^. To extract information from the measured Stokes vector or MM (or part of them), different decomposition methods and parameters were proposed to represent meaningful physical processes, to extract information that could be used in subsequent analysis^52–62^.

### Information extraction from the vector properties of the light beam

**Fig. 5:**
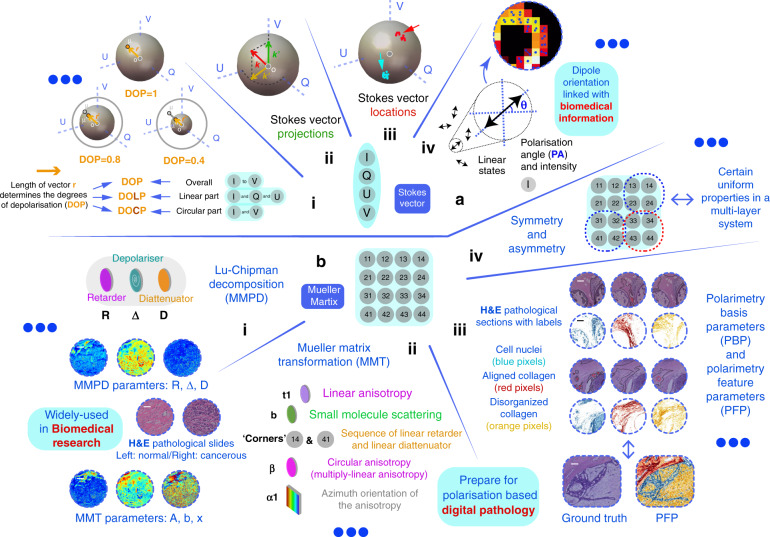
Vectorial information extraction approaches. **a** Stokes vector based approaches: (i) DOP, DOLP and DOCP^2–5^; (ii) Stokes vector projection approach^158^; (iii) Stokes vector location approach^159^; (iv) Dipole orientation differentiation approach. Polarisation angle (PA) and intensity are used as main parameters^154,155^. **b** MM based approaches: (i) MMPD method^52^: diattenuation (D), retardance (R) and depolarisation (Δ) (all of them maintain linear/circular components); and (ii) MMT method^57^: depolarisation (1-b; associated with small molecule scattering), level of linear anisotropy (t1), azimuth orientation of the anisotropy (α1), and more^179^; (iii) PFP method^95^; (iv) Property of symmetry and asymmetry^168,169^. (b(i)) Adapted with permission from ref. ^167^ © The Optical Society. (b(iii)) © [2021] IEEE. Reprinted, with permission, from ref. ^95^

Several parameters can be calculated from the Stokes vector directly (see Fig. 5a (i) and previous section): such as the degree of polarisation (DOP), degree of linear polarisation (DOLP) and degree of circular polarisation (DOCP) of light. For a single uniform light beam, the DOP is 1 for fully polarised, 0 for unpolarised or completely depolarised, and between 0 and 1 for partially polarised. The DOP cannot be larger than 1. Despite containing four elements, a Stokes vector contains fewer than four degrees of freedom due to physical constraints. The Stokes vector can also be considered as an incoherent superposition of a completely polarised part and an unpolarised part^3^. Those parameters have been adopted in different polarimetric applications^16,20,22,23,32,80,81^. The polarisation angle (PA) and intensity of the linear SOP also can be defined, with respect to dipole orientation applications^153–155^ (see Fig. 5a (iv)). For a beam generated via an incoherent light source (such as a LED), the Stokes vectors can be directly added by scalar calculation. Therefore, partially polarised light can be divided into two parts—fully polarised/depolarised components^3^, i.e., **S**_total_ = **S**_u_ + **S**_p_; where **S**_u_ and **S**_p_ represent fully depolarised and polarised components, respectively.

For biomedical and clinical applications, characterising the vectorial properties of the outgoing light with a fixed incident SOP also showed great potential for structure identification^22–24,32^. Wu & Walsh reported that Stokes vector analysis with circular polarised illumination can reveal structural information about tissue^156^. Macdonald & Meglinski showed that turbid tissue can be quantitatively analysed via Stokes vector measurement with an optical clearing technique^157^. Qi et al. proposed a method of Stokes vector analysis for ex vivo porcine tongue, stomach, kidney and other tissues based on circular polarised illumination^158^; the most useful information was provided by circular depolarisation and linear retardance, which can normally be provided via MM decomposition^52,58^ (as Stokes vector projections shown in Fig. 5a (ii)). Kunnen et al. employed Stokes vector detection with circular and elliptical incident SOPs for differentiation between healthy and cancerous lung tissues specifically using a Poincaré sphere illustration^159^ (as Stokes vector locations shown in Fig. 5a (iii)). Note that the circular SOP illumination is especially useful for biomedical analysis, as its effects are independent of the orientation of the anisotropic components that widely exist in biomedical specimens^20–24,32^. What is more, its strong polarisation memory effect with respect to tissue-induced Mie-scattering has also gained attention^160^ (here the memory effect^161^ means that circular polarisation can survive many more scattering events than linear polarisation due to excessive forward scattering, hence it has higher probability to maintain the original information when passing through turbid tissue consisting of Mie-scattering particles that are comparable in size to the wavelength).

### Information extraction from the vector properties of the object

Measurement of the full vector properties of biomedical targets requires illumination with multiple SOPs in combination with multiple analysing SOPs^20–24^. As we have mentioned above, the individual MM elements lack clear physical meanings, or explicit associations with microstructures^20–24^. That is to say, vectorial characteristics of the object, like diattenuation, retardance, and depolarisation are encoded within the MM elements. For a complex optical system (like tissue), each MM element is always associated with more than one polarisation property. Hence, numerous MM decomposition methods were proposed to quantitatively characterise the optical and structural properties of the object^52–59^. One prevalent method is the Mueller matrix polar-decomposition (MMPD) proposed by Lu & Chipman^52^, which has been used and validated in lots of applications for characterisation of biomedical or material samples^58,162–167^ (see Fig. 5b (i)); He et al. put forward the Mueller matrix transformation (MMT) with validations using phantom experiments and Monte Carlo simulations^57^. Based on the MMT concept, more rotation invariant parameters were extracted from the MM and applied to biomedical sample characterisation^167–169^ (see Fig. 5b (ii)); Arteaga and colleagues derived Mueller matrix anisotropy coefficients (MMAC) to describe the level of different kinds of anisotropy for different polarisation systems^56^. Furthermore, other decomposition schemes were also developed, such as MM differential decomposition^55^, symmetric decomposition^60,61^ and Cloude decomposition^62^. Among the various decomposition approaches, different mathematical assumptions need to be made for different applications^52–62^, such as assuming a determined layer sequence of different fundamental polarisation components for a complex object, which in effect simplifies the matrix reciprocity problem^52^. Recently, those methods and related parameters have also been compared quantitatively with each other for the purpose of structural characterisation^170–172^. We can summarise the parameters derived via the above methods: MMPD: diattenuation (D), retardance (R) and depolarisation (Δ) (all of them maintain linear/circular components)^52^; MMT: depolarisation (1-b), level of linear anisotropy (t_1_), diattenuation property (t_2_), level of birefringence (t_3_) and fast axis orientation (x_3_) and more^16,57^; MMAC: horizontal linear anisotropy (α), 45° linear anisotropy (β) and circular anisotropy (γ) respectively with respect to the global anisotropy of the MM^56^.

The MM contains fundamental physical characters like polarisance, diattenuation, retardance and depolarisation (shown in Fig. 2c); however, some concepts like anisotropy can be a combination of several fundamental polarisation processes^16,57^. It is worth mentioning that the depolarisation property—which is used for evaluating a SOP’s disorder, randomness, or uncertainty^3–5^—is also linked with the concept of entropy in polarimetric research^11,173^. While the above parameters are derived from a full MM (4 × 4); Ghosh et al. and Wang et al. also reported works on 3 × 3 MM decomposition methods, related simulations and experiments, with an emphasis on biomedical applications^174,175^. In summary, the decomposed linear depolarisation and linear retardance from a 3 × 3 MM display similar qualitative relationships to the changes with respect to the microstructure of the sample, such as the density, molecule size, and orientation distributions of the scatterers as well as birefringence level of the interstitial medium^174,175^.

The MM decomposition methods all require different assumptions (strong or weak) such as matrix reciprocity, the order that polarisation effects happen in the media, or homogeneity for the tissue analysis^52,58,176–178^. Therefore, their decomposed values are not strictly physically determined, if the assumptions do not hold in reality, which may well be the case, as biological tissue has high spatial complexity^58^. However, extraction through the MM polarisation parameters that have less assumptions and clearer physical meaning is always something to strive for. Several works pointed in such a direction: (1) Gil et al. and Li et al. proposed different polarisation parameters with physical determination via the asymmetric properties of the MM elements^168,169^, by considering assumptions about layer constructions or the presence of absence of specific vector properties such as polarisance or diattenuation; (2) Dong et al. employed a data-driven machine learning technique to fit several polarimetry feature parameters (PFPs) for characterising determined pathological applications, such as detection of the abnormal areas of breast carcinoma and cervical cancerous tissue slices^95^ (see Fig. 5b (iii)); 3) Breaking or restoring the symmetry (see Fig. 5b (iv)), based on analysis of different sub-regions of the MM, to extract determined information of the system is recently gaining interest^179^; The information extraction process is gradually developing from an analytical mathematics approach (equation-based, forward problem), to fitting or observing vectorial semantics/metrics (data-based, or shape/form-based inverse problem).

## Vectorial information analysis for biomedical applications

Polarimetric techniques maintain unique advantages compared with other optical techniques: they can provide extra vectorial information through methods that are compatible with many existing optical systems, such as microscopes and endoscopes^16,24,32,33,92,180^. Much existing biomedical polarimetry research concerns sensing of bio-information in a label-free way without extraneous dyes^16,22,24^. In other areas, polarimetry can be used to characterise the vectorial information of fluorescence dyes, as the dipole orientation of the fluorophore is encoded in the polarisation state of the emitted light^154,155^. The SOP of such emission is always in a linear state; hence the polarisation angle (PA) and intensity of the linear SOP are quantities that can be harnessed, such as in biomedical applications in super-resolution microscopy^153,181,182^. Here we briefly summarise common phantoms used for biomedical polarimetric techniques. These techniques include polarised wide-field microscopy^16,24,183^, polarised light spatial frequency imaging^184^, polarimetric endoscopy^185–190^, spectral light scattering polarimetry^18,82,191–193^, polarised fluorescence spectroscopy^194–196^, polarised confocal microscopy^197^, polarised Raman-spectroscopy^198,199^, polarised super-resolution microscopy^154,155^, polarisation sensitive optical coherence tomography^200–218^, non-diffraction beam polarimetry (such as Bessel beam based)^219^, polarisation-resolved nonlinear microscopy (including second/third harmonic generation)^220–226^, and polarised speckle imaging^213,227^ (several techniques will be mentioned again in the Discussion). The relationship between incoherence and depolarisation of the light should be kept in mind when considering coherence based polarimetric techniques: they are different but related optical concepts. If a polarised coherent beam passing through a scattering medium becomes incoherent, it can result in either polarised light or depolarised light. If after such a medium a polarised coherent beam changes into depolarised, the coherence property may still be maintained. For more details see ref. ^228^. Several of the above techniques have also been adopted in three-dimensional (3D) imaging with signal integrations or sample segmentations^229^. However, numerous existing polarimetry techniques (within the scope of this review) fall into two-dimensional (2D) analysis^23–29^. With the completion of the cutting-edge mathematical interpretations and methodologies (see “Discussion”) there exists of course intriguing scope for further explorations.

In order to understand the interactions between polarised photons and biological specimens, and link the parameters obtained via the Stokes vector or MM with the biomedical microstructural information, a software phantom—Monte Carlo (MC) simulation—was proposed to give plausible explanations for the originality of the observed physical phenomena^45,46^. While biomedical samples are considered as turbid media with complex structures, different fundamental units to mimic the microstructural architecture have been employed: spherical scatterers^35,46^; cylindrical scatterers^41,46^; birefringent intermedia^37–39^, multi-layered geometry^45^ and so on^46^. MC simulations have successfully reproduced most of the important polarimetric characteristic features for biomedical samples^16,230,231^.

### Thin specimens

**Fig. 6:**
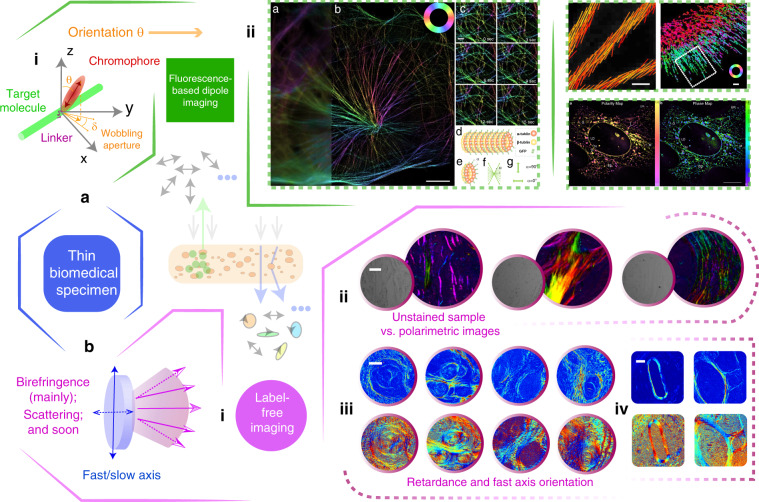
Two categories of the applications enabled via thin specimen polarimetry. **a** (i) Model of the dipole and target molecule with linker-related fundamental mechanism can be found in refs. ^154,155^. (ii) Fluorescence dipole orientation imaging^181,182,233,289^. The polarisation orientation is given as a demonstration. **b** (i) Model of the main properties in thin specimen for label-free polarimetric imaging. (ii) Unstained/polarimetric imaging of human cervical and liver carcinoma tissue samples^32^. (iii) Polarimetric parameters imaging results to distinguish between Crohn’s disease and gastrointestinal luminal tuberculosis tissues^164^. (iv) Polarimetric parameters images of human liver cirrhosis samples in different stages^163^. (a(ii) besides upper-right) Adapted with permission from refs. ^181,182^. CC BY 4.0. (a(ii); upper-right) Adapted from refs. ^233,289^. (b(ii) and b(iv)) Adapted from refs. ^32,163^. CC BY 4.0. (b(iii)) Copyright Wiley-VCH GmbH. Reproduced with permission ref. ^164^

**Fig. 7:**
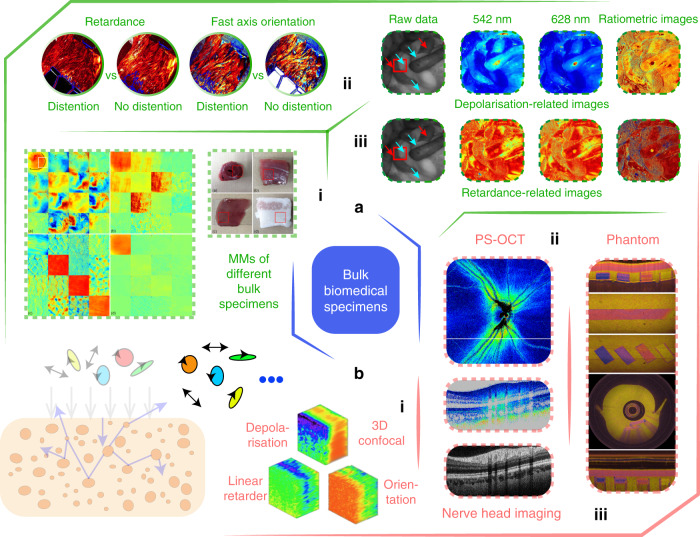
Applications enabled via bulk specimen polarimetry (in vivo and ex vivo). **a** General backscattering-mode polarimetric imaging: (i) MMs and original sample images for several ex vivo bulk specimens^167^. (ii) Polarisation properties of ex vivo bladder tissue^256^. (iii) Polarisation properties using combined wavelength information for in vivo characterisation of rat abdomen tissue^92^. **b** Certain specific backscattering-mode phantoms: (i) Full-depth MM confocal imaging of an unstained rat cornea^265^. (ii) PS-OCT imaging for a nerve head, which is conducted under the condition of in vivo eye imaging^34^. (iii) A birefringent phantom designed for bulk tissue research^270^. (a(ii)) Adapted with permission ref. ^256^. CC BY 4.0. (a(i), a(iii), b(i), b(ii) and b (iii)) Adapted with permission from refs. ^34,92,167,265,270^ © The Optical Society

Specimens and their mimicking phantoms can be thin or bulky, which also, in general, determines the configurations of the biomedical polarimetry. A transmissive geometry is used for the thin cases (see Fig. 6) which are less scattering, thus most of the incident photons would be transmitted. A backscattering geometry (see Fig. 7) is preferred for the bulk cases (ex vivo and in vivo) which are highly scattering and depolarising, thus most of the incident photons would be backscattered. There is no clear boundary between what constitutes thin or bulk tissues. Indeed, intermediate or mixed states can exist, for which both the transmission and backscattering photons can be detected simultaneously^16,22,23,152^. In response to the beginning of this section, biomedical polarimetry can be used in labelled or label-free measurement; Fig. 6 gives a summary for two types of the use of thin tissue polarimetry.

For label-based direction, polarimetry has found use in scientific applications, such as biomedical microscopy^16^. The vectorial information of the dipole emitters is encoded in the SOP of the detected light^153,154^. The dipole orientation (and the fluorescence intensity) polarimetric detection technique plays an important role in thin biomedical sample analysis: e.g., in fluorescence polarisation microscopy (FPM)^194,195,232,233^; FPM can be used to study the nuclear pore complex subcomplexes and the relative orientations^234^, or be used to study different types of cytoskeleton such as actin, myosin, kinesin, microtubule and septin—those closely related with the performance of the dipole behaviours^235–239^—enabling research such as ATP and ADP binding^237^. Advanced research has been adopted in super-resolution imaging harnessing fluorescent dipoles via polarised illumination, with applications such as revealing heterogeneity and dynamics of subcellular lipid membranes^181,240,241^. These fluorescence anisotropy properties also belong to the fundamental polarisation properties that are encoded in the MM.

For label-free biomedical polarimetric research, especially in clinical/pathological related topics, cancerous tissues detection is an important application^22–24^. In the past decades, such polarimetric techniques have assisted the diagnosis of various cancerous tissues, such as human skin cancer^242^, cervical cancer^243–246^, colon cancer^166,247–250^, liver cancer^163,251^, breast cancer and gastrointestinal cancer^93–95,252^. A typical bio-information analysis of polarimetric data is for quantitative evaluation of the fibrosis process among different stages of cancer development^94,163^. Beside the degree of fibrosis that can be quantified via biomedical polarimetry, the distribution of features in the fibrous regions also can serve as another characteristic parameter to assist the pathological diagnosis; this distribution can be readily extracted via polarisation information^164,165,171^. Intuitively, such structures contribute intrinsic birefringence mainly affecting the fourth row and fourth column of the target MM^16^. A good demonstration in ref. ^164^ shows how polarimetric textural mapping of retardance properties can distinguish between Crohn’s disease and gastrointestinal luminal tuberculosis tissues (see Fig. 6b). Some thin specimen phantoms, as found in ref. ^152^, target the fundamental understanding of the constitution of certain biomedical specimens, such as using nanoparticles or microspheres. Moreover, polarimetry has recently been applied to other diseases detections including Alzheimer’s disease and bladder outlet obstruction^24,253,254^.

### Bulk specimens

Polarisation techniques can help improve the image contrast of the superficial layers of tissues by eliminating multiply scattered photons from the deep layers^20–24^. The previous literature shows that more than 85% of cancers originate from the superficial epithelium, which means that polarisation imaging methods have great potential in screening and identifying cancer at an early stage^255^. This would be specifically useful for in vivo clinical diagnosis, such as for minimally invasive surgery (MIS)^24^. Measurements in ex vivo thin tissue can use a transmissive geometry, whereas ex/in vivo bulk tissue detection would need backscattering configurations. Figure 7 gives a brief demonstration of certain current research topics related to bulk tissue polarimetry.

For polarimetric bulk tissue research, ex vivo detection plays an important role^22–24^. For example, collagen fibres, which widely exist in tissues and organs such as tendons, skin and bladder (from porcine, swine, lobster, calf or other animals^156,256–259^), skeletal and myocardial muscle fibres^260–262^, and elastin fibres are widely used due to their linear birefringence properties^20–23,164^. The alignment directions of all such fibrous structures are also linked with the fast axis orientation of the generated linear birefringence^16,165,171^. Furthermore, the scattering of bulk media is also studied via the extracted depolarisation^16,167,263^. The retardance and depolarisation related properties are the dominant parts of the vectorial properties of bulk tissues, as the magnitude of diattenuation for majority of tissue is typically very small^158^, with several exceptions like skeletal and myocardial muscles^167^ (see Fig. 7a (i)). Previous research analysing muscle tissue^264^ showed lower retardance compared with tendon tissue, owing to the cellularity of these tissues. Sections of the bulk myocardial fibre tissues showed two circularly aligned ring-shaped fibrous structures (see Fig. 7a (i)), revealing their anisotropic properties^167^. The different anisotropic vectorial information obtained from polarimetric measurements can be very helpful for the discrimination and identification of different fibrous structures in tissues^164,165,167^.

While ex vivo studies are mainly oriented towards fundamental research^24,152,171^ (e.g., understanding the vectorial properties characterisation; see Fig. 7a (i), (ii), b (i)), in vivo bulk tissue polarimetry is geared towards applications^24,256,265^. Typical backscattering-mode polarimetry includes polarisation endoscopy^24^, reflection MM microscopy^16,266^, MM colposcopy^180,267^, wide-field handheld polarimetry^16^, and PS-OCT^268^ (see Fig. 7a (iii), b (ii)), targeted to clinical diagnosis in vivo. As a promising in vivo, label-free diagnostic tool, polarisation endoscopic imaging has been implemented inside rat abdomen, revealing the small bowel, stomach, liver and fat with different polarisation characters^92^. Recent work also includes development of several different types of MM endoscope^188–190,256^, and extension into the spectral domain (with certain fixed wavelengths)^92^ (see Fig. 7a (iii)). PS-OCT^200–218^ is specifically used for in vivo ophthalmic imaging, where polarimetric data accompanied with clinical analysis has been demonstrated, for retinal imaging^268^ (see Fig. 7b (ii)). Other types of bulk tissue analysis, such as human lung cancerous tissue^159^ and skin tissue^269^, show good prospects for future clinical diagnosis^16,22–24^.

While thin samples can feature multiple-scattering process, such processes are of course more significant in bulk samples^20–22^. Considering the intriguing scope of the polarimetric technique for in vivo clinical diagnosis, beside the simulations, there is a need for complex phantoms, such as those exhibiting birefringence (see Fig. 7b (iii)) or depolarisation, to establish reliable processes for investigation of complex scattering mechanisms^270,271^. A recent review has summarised various phantoms for both thin and bulk samples^152^. Microspheres, silicon-based phantoms, nanoparticles, cylindrical scatterers, and birefringent/dichroism films^35,158,270,272^ have all been employed in various validations. In vivo biomedical polarimetry and its related applications clearly offer a large space for future exploration.

## Directions for advanced biomedical polarimetry and future prospects

**Fig. 8:**
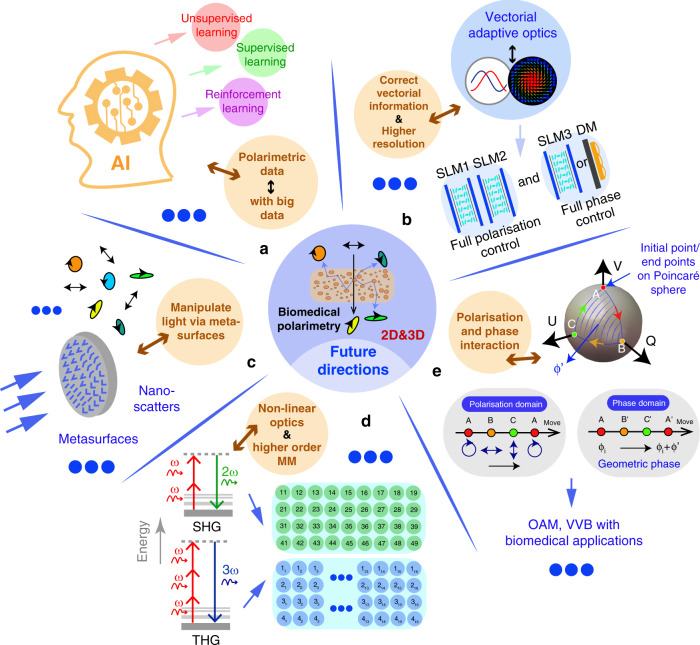
Future directions for biomedical polarimetry. **a** Combination with machine learning techniques and big data. **b** Combination with vectorial adaptive optics. **c** Combination with metasurface based techniques. **d** Combination with nonlinear techniques, such as SHG and THG, in which a high order MM is required. **e** Combination with absolute phase information, such as geometric phase, with potential applications related with orbital angular momentum (OAM) and vector vortex beam (VVB) manipulations

Biomedical applications of polarimetry have attracted substantial attention. We hope this short review paper gives readers a general overview from fundamental polarisation concepts, through polarimetric techniques, to recent biomedical and clinical applications^7,16,20–24,29,34,63,79,152^. In addition to the summaries of recent research trends explained above, we provide here some further perspective on prospects in this application area, considering the use of polarimetry in a multimodal combination with other advanced technologies (see Fig. 8 for a summary).

Firstly, the fast development of machine learning (ML) is clearly going to have an impact on this field^95,273,274^. Such data-driven techniques may pave new directions for biomedical polarimetry, either through improving the quality of polarimetry (such as overcoming the numerous sources of error) or through enhanced information extraction^68,95^. One possibility is to use low-resolution information to reconstruct high-resolution patterns (following the spirit of works such as refs. ^275,276^). Secondly, while ML is geared towards improving the information processing aspects of polarimetry, new adaptive optics techniques can be used to extend the capabilities of polarimetry through full vectorial beam control. This could enable enhanced polarisation imaging resolution physically via beam shaping and compensation of polarisation errors^277–280^. Thirdly, the emerging techniques based on metasurfaces—subwavelength arrays of nano-scatterers that can modify polarisation—have been adopted for polarimetry^281^, as well as for 3D polarisation control^282^. Such developments may bring new opportunities for advanced biomedical polarimetry, such as forming compact vectorial sensors^24,283^ for deep tissue information extraction. Fourthly, second harmonic generation (SHG) and third harmonic generation (THG) based 3D MM techniques have been proposed^284–287^. These are described by extended MMs that are more complicated than 4 × 4 MMs used for linear scattering (4 × 9 and 4 × 16 elements, respectively, for SHG and THG)^284,285^. For these methods, further advanced information extraction and analysis approaches are of course intriguing. Finally, the intensity and wavelength have been utilised together with polarisation in polarimetry for a long time. However, the absolute phase information—especially geometric phase-related techniques^8,10^—may again open windows for new biomedical polarimetry approaches with multi-modal performance.
